# Demand and influencing factors of “Internet + Traditional Chinese Medicine” home nursing service for older adult patients with chronic diseases: a mixed research perspective

**DOI:** 10.3389/fpubh.2023.1271082

**Published:** 2023-10-17

**Authors:** Xinghuan Wang, Jinyan Chen, Meiqin Feng, Miaoqing Zhuang, Jiayi Wang, Luyu Zhang, Yue Liu, Hongfang Chen

**Affiliations:** ^1^School of Nursing, Shaanxi University of Traditional Chinese Medicine, Xianyang, China; ^2^Personnel Department, Shaanxi Provincial Hospital of Traditional Chinese Medicine, Xi'an, China

**Keywords:** chronic disease, internet, Chinese medicine, home care, needs

## Abstract

**Background:**

In the context of global aging, the characteristics of chronic diseases seriously affect the quality of life of older adults. It is urgent to carry out continuous nursing basis for older adult patients with chronic diseases. In view of the remarkable efficacy of Chinese medicine in the treatment of chronic diseases, this study may help to understand the demand for “Internet + Chinese medicine” home care service and its influencing factors of older adult chronic disease patients, and to provide a reference basis for improving the quality of life of the older adult chronic disease population.

**Methods:**

This is a mixed study. The quantitative study adopted the convenience sampling method, and a total of 308 patients in a third-grade hospital in Shaanxi Province were investigated by general data questionnaire, traditional Chinese medicine service demand questionnaire, traditional Chinese medicine knowledge questionnaire, older adult Chinese medicine attitude questionnaire, and home care demand questionnaire from March to April 2022. In the qualitative study, semi-structured interviews were adopted, and patients were interviewed until the content was saturated. Colaizzi analysis method was used to analyze and summarize the topic of the interview data.

**Results:**

308 valid questionnaires were collected, and the patients scored (58.42 ± 17.16) on the demand for TCM nursing services, (59.86 ± 11.54) on the knowledge of TCM, (73.03 ± 9.11) on the attitude toward TCM, and (136.84 ± 46.39) on the demand for home care. The results of multiple linear regression showed that learning about the nursing service pathway, knowledge of general knowledge of Chinese medicine, and attitude toward Chinese medicine among the older adult and home care demand were the influencing factors of the demand for Chinese medicine nursing services for older adult patients with chronic diseases (*p* < 0.05). The results of the in-depth interviews were summarized into three themes: facilitating factor, hindering factor, and the “Internet + Chinese medicine” multiple needs of home care.

**Conclusion:**

Older adult patients with chronic diseases have a high intention of home care demand and they are affected by multiple factors. Consequently, the actual demand situation of older adult patients with chronic diseases should be used as a guide to provide directed and diversified Chinese medicine home care services to meet the individualized needs of the older adult.

## 1. Introduction

Population aging has become a global problem and exerted heavy burdens on the health care system and society. By 2030, the world is likely to have 1 billion people aged 65 years or older, accounting for 13% of the total population (1, 2). As the world’s most populated country, China is facing a major challenge due to the aging population. China’s aging population is estimated to increase at a rate of 5.96 million per year from 2001 to 2020 and then 6.2 million per year from 2021 to 2050, and is expected to exceed 400 million by 2050, accounting for 30% of its total population (3). According to the survey, the death of chronic disease population accounts for 89.82% of total deaths (4). With the increase of average life expectancy, the frequent occurrence of chronic diseases is increasing year by year, such as diabetes, cardiovascular disease, chronic obstructive pulmonary disease, and cancer, which will become an important challenge for the aging society (5, 6).

Older people are vulnerable and have complex health care needs, especially during the transition from hospital to home, which is a vulnerable time for older people. In addition to social reintegration and family reintegration, there is a need to find new care options to maintain the disease (7). Chronic diseases are long-lasting, slow to progress, and often associated with multiple states. Older adults with chronic conditions have ongoing complex care needs that require multiple care settings. Their lifestyle is characterized by frequent changes in health, high rates of rehospitalization, and the long-term involvement of family and health care providers in their care.

With the advancement of wearable health devices, cloud computing, mobile technologies, and Internet of Things, mobile health (m-Health) is rapidly developing and shows a promising future in the management of chronic diseases. Its advantages include its ability to improve the quality of care, reduce the costs of care, and improve treatment outcomes by transferring in-hospital treatment to patient-centered medical treatment at home. M-Health could also enhance the international cooperation of medical providers in different time zones and the sharing of high-quality medical service resources between developed and developing countries (8). Home nursing service is an important part of extended nursing services, which refers to that the community nurses come to the patient’s home to provide continuous and systematic basic medical care services under the premise of medical advice. Studies have shown that home care services for older adult patients, taking into account both physical and psychological care, can reduce the burden of disease, solve or improve the health problems of older adult patients, and promote healthy aging. At the same time, it can also make up for the lack of child care and reduce the burden of family life. Therefore, the demand for home care services for older adults gradually increasing, and home care services are imperative.

However, the uneven distribution of caregivers and critical health care workforce shortages are major obstacles to improving disease outcome. Most older adult patients with chronic diseases require long-term out-of-hospital medical care, so there is a greater demand for out-of-hospital care (9, 10). Home care is an extended service after discharge, mainly through the intervention operation of the professional team of home nursing, which can provide highly targeted nursing services for older adult patients with chronic diseases (11). Older adult patients with chronic diseases have low immunity and many underlying diseases, according to the previous health care work more emphasis on care and maintenance during hospitalization, while ignoring the basic health care of patients after discharge. Tele-medicine, e-health, and m-health scenarios can improve health outcomes, quality of life, and well-being and facilitate functional patient empowerment and engagement.

Internationally, the United States, Canada, Australia, and Japan have developed relatively mature home care service models with their own unique characteristics (12). These countries have similar home care service processes, but each has its own characteristics in terms of home care providers and service content. Home health agencies in the United States are the only Medicare-certified providers allowed to provide skilled nursing care for acute, chronic, and rehabilitative conditions in people’s homes, with more than 12,300 home-based agencies participating in Medicare in 2015 and approximately 3.5 million Medicare beneficiaries receiving services (13). Most regions of France have begun piloting the inclusion of informal caregivers and social workers from informal healthcare organizations into the assistance system to assist healthcare workers (14). In developed countries abroad, the place of treatment has shifted from hospitals to community-based outpatient clinics and homes, and home care for cancer pain is undertaken by specialized home care teams or agencies (15). Japan promotes a community-based integrated care system, provides financial support through long-term care insurance, and establishes home care visiting stations to provide a variety of home care services to discharged patients (16). In Poland, home pain management nurses, hospital nurses, professional family caregivers, and family members are involved in home care, taking on tasks such as health education, medical counseling, promoting rehabilitation, and rational allocation of medical resources (17). From foreign experience, scientific allocation of home care services and human resources can improve patients’ quality of life and satisfaction and promote efficient utilization of limited health resources (18, 19).

A prior literature review also found that the implementation of various home care models in the international arena is a specific model of home care under a unique policy and health insurance system and has its own advantages and disadvantages. Models in which private and social insurance pays and private agencies provide services (United States model). A model in which the government pays and private agencies provide services (the Canadian model). A model in which the government and the individual jointly pay for and diversify the service providers (Australian model). The model of insurance payment and diversified service providers (Japanese model). However, the health care policy and medical insurance system implemented in China are very different from those of other countries, and the contents of our home care programs are scattered in both theory and practice, without forming a scientific and unified model of home care. Therefore, it is not possible to completely copy the foreign home care model, but to utilize the experience of each country to establish a TCM home care model to meet the specialized care needs of home patients, to reduce the burden on the family and society, and to improve the quality of life of the patients.

The innovation of this study is the effective combination of medical association, “Internet + Chinese medicine” and home care. Older adult patients with chronic diseases, as the disseminator of Chinese medicine culture, utilize the “Internet + ” technology, and gradually spread the unique advantages of Chinese medicine among the community patient groups. Utilizing the “Internet Plus” technology, the unique advantages of Chinese medicine are gradually widely disseminated among the community patient groups, thus promoting the sustainable development of the Chinese medicine cause. Adopting a combination of qualitative and quantitative methods, we have gained an in-depth understanding of the needs of older adult patients with chronic diseases for TCM home care services. Through the penetration and integration of TCM technology and home care, we can help patients solve the problems of lack of self-care knowledge and insufficient nursing support after discharge from hospitals in a timely manner, so as to ensure the continuity and timeliness of the service and effectively improve the quality of life of older adult chronic disease patients.

The convenience and effectiveness of “Internet + Traditional Chinese medicine” nursing shows its unique superiority in prevention and treatment, convalescence, rehabilitation, etc. It has outstanding intervention effect on chronic diseases of older adults and can break through the limitations of traditional nursing service mode, and its nursing mode has been recognized and advocated by many scholars (20). However, “Internet + TCM” is currently limited to online appointment registration, patients still need to go to the hospital by themselves, for people still need to improve the convenience, with the concept of continuous nursing in recent years, making “TCM + home service” possible. The unique efficacy of TCM in the chronic disease prevention and treatment system has become a necessary way to consolidate primary health services, and the expansion and extension of the nursing service field has been urgently needed (21). As there are fewer studies on online home care services of TCM, this study integrates quantitative and qualitative findings to explore the current situation of demand for home care services of older adult patients with chronic diseases and the factors influencing it, and to provide reference for the construction of the “Internet + TCM” home care services, with a view to providing reference for the management of chronic diseases and the development of home care services of Chinese medicine.

## 2. Materials and methods

### 2.1. Research object

This study selected 308 older adult patients with chronic diseases who met the inclusion and exclusion criteria in seven departments, including geriatrics, cerebral diseases, cardiology, and endocrinology, of Shaanxi Provincial Hospital of Traditional Chinese Medicine from 2022-03-01 to 2022-04-30 as the subjects of the survey. Purposive sampling method was used in the interview part to determine the final inclusion of the interview subjects in this study based on the principle of reaching saturation of information. A total of 25 patients were finally interviewed.

Inclusion criteria: ① Age ≥ 60 years old. ② Medically diagnosed with Chronic diseases such as stroke, coronary heart disease, diabetes, etc. ③ History of the disease is more than 2 years. ④ Score of self-care ability >60 points. ⑤ Ability to read, communicate, and understand, and be able to give informed consent.
Exclusion criteria: ① Combined with malignant tumors or serious organic diseases.② Speech and language disorders and serious audio-visual disorders.

#### 2.1.1. Sample size calculation

Based on the sample size calculation formula of cross-sectional study:


$$n={\left(\frac{u_{\alpha /2}\sigma }{\delta }\right)}^{2}$$

Calculated by “the demand for home care services for older adult patients,” (significance level = 0.05, Z = 1.96, refer to the related literature *p* = 58.9%, *q* = 1−*p*, and *d* = 0.1*p*) the minimum calculation value of the sample size is about 268 cases. The minimum calculated value was about 268 cases and considering 15% invalid questionnaires, the study finally selected 308 older adult patients with chronic diseases for the survey. The study was approved by the hospital ethics committee.

### 2.2. Research methods

#### 2.2.1. Quantitative research

According to the purpose of this study, a general data questionnaire was designed by searching a large number of domestic and foreign literatures on home nursing, combining China’s national conditions and the characteristics of chronic diseases in older adults. The content of the questionnaire was verified by six experts, and a preliminary survey was conducted on 50 patients before the formal survey. According to the problems found in the preliminary survey, the questionnaire was further screened and revised, and the following five scales were finalized.

##### 2.2.1.1. General information questionnaire

Sixteen items including age, sex, residence, education level, degree of understanding of the contents of the **“**Internet + TCM**”** nursing home service approach, how to learn about the **“**Internet + nursing home service**,”** and what are the concerns about carrying out the **“**Internet + TCM**”** nursing home service etc.

##### 2.2.1.2. Questionnaire on the demand for Chinese medicine services

The scale was compiled by Du (22) to assess the older adult’s demand for TCM characteristic services, with a total of 17 items. The degree of demand is mainly defined according to the number of items of TCM characteristic services needed by older adults: no need at all, no need for TCM characteristic services; Not very need, need a traditional Chinese medicine characteristic service; General need, need 2–3 TCM characteristic services; For those in need, 4–5 TCM characteristic services are needed; Very need, need more than five TCM characteristic services. According to Likert’s five-point scale, it is divided into very needed (five points), needed (four points), average (three points), less needed (two points), and not needed (one point). The higher the score, the higher the demand. In this study, the Cronbach’s ɑ coefficient was 0.976, half reliability was 0.937, and a KMO value was 0.945, *p* < 0.01.

##### 2.2.1.3. Questionnaire on general knowledge of Chinese medicine

This questionnaire was developed by Ren (23). The objective is to understand the general knowledge of traditional Chinese medicine in older adults and explore the relationship between it and the attitude toward traditional Chinese medicine. The content of the questionnaire includes the knowledge of 10 aspects such as the basic characteristics of traditional Chinese medicine and basic diagnosis and treatment methods. Each item is scored on a Likert four-scale according to the degree of knowledge, where those who choose “fully know” and “basically know” are recorded as knowing, and those who choose not know are not aware. Each item was scored with 2.5–10 points respectively, and the total score of the item was 100 points. The final score was analyzed by SPSS project, and the score was arranged in descending order, with the front and back 27% as high and low level, and the middle 46% as medium level. In this study, the Cronbach’s ɑ coefficient was 0.894, with a half reliability of 0.701, and a KMO value of 0.886, *p* < 0.01.

##### 2.2.1.4. Attitude scale of traditional Chinese medicine for older adults

This scale was compiled by Liu (24) to understand the attitude of older adults towards traditional Chinese medicine. The scale was developed by item screening, factor load detection, factor number determination, and SPSS classification processing. It was composed of three dimensions, namely “cognition of current situation of Chinese medicine,” “emotional belief of Chinese medicine,” and “tendency of seeking medical treatment with Chinese medicine,” among which the dimension of “cognition of current situation of Chinese medicine” contained 10 items, and was evaluated from the perspective of older adult people’s perception of Chinese medicine service level. The “Emotional Beliefs of Traditional Chinese Medicine” contains 12 items, which are assessed by older adult’s trust in the clinical efficacy and price advantages of traditional Chinese medicine. The “Tendency to seek medical Treatment with Traditional Chinese Medicine” consists of three items, which mainly assess the degree of propensity to traditional Chinese medicine when older adults are sick themselves and their family members. There are a total of 25 items, ranging from agree to disagree, with scores ranging from 1 to 4 points. The cognitive dimension of TCM status is negative, and the total score is 100 points. In this study, the Cronbach’s ɑ coefficient was 0.827, half reliability was 0.658, and a KMO value was 0.831, *p* < 0.01.

##### 2.2.1.5. Questionnaire on home care needs of patients with chronic diseases

The questionnaire was compiled by Tian (25) to analyze the home nursing needs of discharged patients with chronic diseases, including clinical nursing (12 items), health guidance (13 items), rehabilitation nursing (five items), life nursing (10 items), and psychological nursing (2 items), with a total of 42 items. Likert 3 scoring method was adopted for the questionnaire, which was divided into need (five points), indifferent (three points), and do not need (one point), with a total score of 210 points. The score value obtained by all items in each aspect is summed and the average score is taken as the score value of this aspect (because the number of items contained in each aspect is different, the average value of items is taken to represent the score value of this aspect); The score values obtained by all items are summed and averaged as the score value of total demand. The higher the score, the higher the demand for home care. In this study, the Cronbach’s ɑ coefficient was 0.973, half reliability was 0.928, and a KMO value was 0.886, *p* < 0.01.

#### 2.2.2. Qualitative research

Based on the literature review, the interview outline was initially formulated according to the research purpose. Combined with experts’ opinions and after pre-interviewing three older adult patients with chronic diseases, the research team repeatedly discussed and revised the final interview outline as follows:

Please tell us about your understanding and willingness to accept the “Internet + Chinese medicine” home care service. (2) What are the benefits to you if the “Internet + Chinese medicine” home care service is carried out? (3) What difficulties or problems will you encounter if the “Internet + Chinese Medicine” home care service is launched? (4) Please tell us what medical and nursing services and TCM nursing techniques you would like to receive in the development of “Internet + TCM” home nursing service for your disease? (5) What needs or suggestions do you have for the development of “Internet + TCM” home care service?

#### 2.2.3. Data collection

##### 2.2.3.1. Quantitative study

The survey was implemented in a one-to-one on-site format. Prior to the survey, a trained investigator explained the purpose and significance of the study to the patient in detail. They sign informed consent questionnaires, which are handed out to patients in person by investigators who can assist patients who cannot fill out the questionnaires themselves. Check immediately after filling, fill and correct in time.

##### 2.2.3.2. Qualitative research

Semi-structured interviews were used, in which the researcher explained the purpose and content of the interview to the interviewer, and audio-recorded the content of the interview with his informed consent. Each interview lasted about 30 min, and non-verbal behaviors such as facial expressions and body movements were recorded in a timely manner. Within 24 h after the interview, the audio recordings were promptly transformed into written texts, and the content of the subsequent interviews was adjusted according to the situation of each interview.

#### 2.2.4. Statistical analysis

##### 2.2.4.1. Quantitative research

Excel was used to input the questionnaire and establish the database. SPSS 26.0 was used for statistical analysis. Describe categorical variables in terms of frequency and percentage. Normal test was performed on all scale data. If normal distribution was followed, the mean and standard deviation were used to describe continuous variables; otherwise, the median and interquartile distances were used. Independent sample t and one-way ANOVA were used to analyze the demand for traditional Chinese medicine nursing services. Data that met normal distribution and homogeneity of variance were analyzed by using two-independent sample *t* test and ANOVA. Data that did not meet normal and homogeneity of variance were analyzed by non-parametric test. Mann–Whitney U test and Kruskal-Wallis H test were used. Pearson or Spearman correlation was used to analyze the correlation between TCM nursing service demand, TCM knowledge, TCM attitude, and home nursing demand of chronic disease patients. Taking TCM nursing service demand score as dependent variable, the results of univariate analysis and correlation analysis were put into the equation, variables with significant differences were selected as independent variables, and the main influencing factors were determined by multiple linear stepwise regression analysis. All the above tests were conducted by bilateral test, the test level was *a* = 0.05, and *p* < 0.05 was considered to be statistically significant. Cronbach’s Alpha and KMO were obtained through the reliability analysis and validity analysis of the four scales.

##### 2.2.4.2. Qualitative research

A semi-structured interview was used, in which the investigator told the interviewer about the purpose and content of the interview and recorded it with their informed consent. Each interview time was approximately 30 min. After the interviews, the interviews were converted into text verbatim and sentence-by-sentence within 24 h by two researchers. The information was organized and analyzed using the colaizzi seven-step analysis method (26). The data were managed and analyzed with the help of Nvivo 12 Plus software.

#### 2.2.5. Ethical approval

All participants were informed of the purpose of the study and obtained written informed consent prior to the survey and interview. The participants voluntarily chose to participate in this study and were free to drop out. The electronic information submitted was anonymous, and only the researchers had access to data. The Ethics Committee of the Shaanxi Provincial Hospital of Traditional Chinese Medicine, Shaanxi Province, China approved this study.

## 3. Results

### 3.1. Quantitative findings

#### 3.1.1. General information of respondents and one-way analysis of factors

##### 3.1.1.1. General information of survey respondents

A total of 308 questionnaires were distributed in this study, and 308 valid questionnaires were recovered, with a valid recovery rate of 100%. The age of these 308 older adult patients with chronic diseases was 60–93 (71.06 ± 7.30) years old; the highest concern about the development of Internet + nursing home service was the high cost of the service 144 (46.8%); and the willingness to choose nurses’ home service was 227 (72.7%), uncertainty 63 (20.5%), and unwillingness 21 (6.8%). The general information of the survey respondents is shown in Supplementary Table 1.

#### 3.1.2. Older adult patients with chronic diseases need for Chinese medicine nursing service, Chinese medicine awareness, Chinese medicine attitude, and home nursing service demand score

The demand score of TCM nursing service was (58.42 ± 17.16) points. The Chinese medicine knowledge score was (59.86 ± 11.54). The score of TCM attitude was (73.03 ± 9.11), the score of TCM status cognition dimension was (24.18 ± 3.97), the score of TCM emotional belief dimension was (39.86 ± 5.60), and the score of TCM tendency to seek medical treatment dimension was (8.99 ± 2.31). The score of home nursing demand was (136.84 ± 46.39), the score of clinical nursing dimension was (36.62 ± 15.10), the score of health guidance dimension was (50.30 ± 14.93), the score of rehabilitation nursing dimension was (18.08 ± 7.03), and the score of life nursing dimension was (25.60 ± 12.76). The psychological nursing dimension score was (6.23 ± 3.16). Through the items of therapeutic effect, selection tendency, frequency of use, and cognition of Chinese medicine by older adults, we have a good practical use and feeling in the Chinese medicine diagnosis and treatment of older adult patients with chronic diseases.

#### 3.1.3. Multiple regression analysis of the demand for TCM nursing services for older adult patients with chronic diseases

Stepwise multiple linear regression analysis was carried out with the total demand for Chinese medicine nursing service of older adult chronic disease patients as the dependent variable, and the variables with statistically significant differences in the univariate and correlation analyses as the independent variables. The results showed that the facilitating factors were the attitudes of older adults toward Chinese medicine, the obstructing factors were the awareness of the current status of Chinese medicine and digital literacy, and the demand factors were the degree of demand for home care services, which were the influencing factors of the demand for Chinese medicine care services for older adult patients with chronic diseases (*p* < 0.05). The variable assignments are shown in Table 1, and the results of regression analysis are shown in Table 2.

**Table 1:** Assigns and variables.

Variables	Assigns
The number of chronic diseases	1 species =1, 2 species =2, 3 species =3, and more than 3 species =4
Whether the caregiver can meet the care needs	Fully satisfy = 1, Can basically satisfy = 2, Cannot satisfy = 3, and Cannot satisfy at all = 4
Knowledge of home care level	Do not understand = 1, Have a certain understanding = 2, and Have experienced the home care service = 3
Know about internet + Nursing home care: newspaper television, mobile computers, and others	Yes = 1, No = 0
There are concerns about nursing door-to-door service, such as low personal safety, high service cost, high risk of nursing operation, large nurse workload, and other	Yes = 1, No = 0
Whether you would like home care services	Willing = 1, uncertain = 2, and unwilling = 3

**Table 2:** Results of multiple linear regression analysis (*n* = 308).

	Regression coefficient	Standard error	Standard coefficient	*T* value	*p* value
(Constant)	−15.646	4.834		−3.237	0.001
Questionnaire on home care needs of patients with chronic diseases	0.254	0.013	0.686	20.085	<0.001
Questionnaire on general knowledge of Chinese medicine	0.262	0.047	0.176	5.558	<0.001
Attitude scale of traditional Chinese medicine	0.304	0.062	0.162	4.928	<0.001
Know about internet + Nursing home care: the mobile phone or computer	3.951	1.337	0.094	2.956	0.003
Know about internet + Nursing home care: the newspaper or television	3.493	1.366	0.079	2.557	0.011

*R* = 0.845, *R*^2^ = 0.714, correction *R*^2^ = 0.709, *F* = 45.159, *p* < 0.001.

### 3.2. Qualitative findings

#### 3.2.1. Theme 1: facilitating factors

##### 3.2.1.1. Stronger willingness to participate

Most of the patients expressed their willingness to participate in the “Internet + TCM “home care service model in the interviews. N6: “Now sometimes the bed is not available, you have such good conditions and good services, of course I am willing.” N16: “Acceptable, I feel quite good, for those who cannot move freely, cannot take care of themselves, or you are not very convenient, like this situation home care is very good.” N1: “Then surely there is a network form of that would be better, there are people who value a little bit, a little bit of economic stability, that’s surely a good thing, TCM home care, this trend is good.”

##### 3.2.1.2. Perceived usefulness

Interviewed patients generally agree that “Internet + Chinese medicine” home care has the characteristics of convenience and professional service, in addition, patients believe that Chinese medicine care technology has better advantages and effects on chronic diseases. In addition, “Internet + TCM” home care can provide patients with psychological care. N5: “I think it’s good, actually it’s very convenient for the patients, it’s better for the people.” N10: “It is inconvenient to queue up at the hospital. We should develop Chinese medicine home treatment, I think it’s good.” N19: “Our own massage is definitely not as good as a professional one.”

#### 3.2.2. Theme 2: obstacles factors

##### 3.2.2.1. Cognitive limitations

The interviewed patients have only a superficial understanding of the “Internet + Chinese medicine” home care service model, and have no in-depth knowledge of it. N9: “I have only seen it in the media, on TV, on the bulletin boards, but I have not knew anything else about it, so my knowledge of it is quite shallow.” N11: “If you look at people like us who can move on our own, we do not usually need home care. Those who generally need home care are those who lie in bed and are basically immobile.” N22: “I’ve heard of it by chance, but I think it’s quite far away from me, and I have not taken it into consideration. Whether it can be involved depends on the financial situation of the family.”

##### 3.2.2.2. Low level of digital literacy

Introducing the “Internet + TCM” home care model to patients before the interview. Older adult patients with chronic diseases have relatively less access to cell phones and the Internet, and their ability to use cell phones is limited, resulting in a lower level of digital literacy, which hinders the willingness of some patients to participate in the “Internet + Chinese medicine” home care service. N4: “I cannot go on the Internet, but I usually come to the hospital when it’s time. It’s hard for me to understand some of the online operations, no way.” N5: “The hospital sometimes has some medical information on the public number, but I do not have to add these public numbers, I do not know how to do this.”

##### 3.2.2.3. Cost of services and geographical factors

The cost of diagnosis and treatment, geographical and transportation barriers, and the lack of institutions and equipment make it difficult and challenging to carry out “Internet + TCM” home care services at this stage. N8: “This is because the older adult people have to consider the issue of cost, and participation in health insurance must be considered.” N21: “This and some of the country’s policies are related to various aspects, for example, now like family wards or community hospitals, the government has to do that.” The community does not have these facilities yet, this service model, first of all this institution has to be available. N12: “It would be better for urban or urban–rural areas. Because people live more sparsely, the population is not so full, and in addition people are more dispersed, and the medical conditions are limited in rural areas.”

#### 3.2.3. Theme 3: multiple needs of “Internet + TCM” home care

##### 3.2.3.1. Demand for the provision of diversified care programs

“Internet + Chinese medicine” home care needs to provide Chinese medicine nursing appropriate technology, basic care, Chinese and Western medicine combined care, health guidance, and other comprehensive and systematic care programs. N21: “For example, some simple physical therapy, infusion, these are all possible.” N8: “The best is more complete, incorporating both conventional Western therapy and Chinese medicine.” N12: “Look at the treatment of disease, it is comprehensive treatment, you cannot say that Western medicine is good and take away Chinese medicine, Chairman Mao has said that we should combine Chinese and Western medicine.” N9: “I think if I can really do it, in fact, according to my current situation, my problem is not very serious, if I really encounter some problems, I can consult you.”

##### 3.2.3.2. Dialectical care and the need for quality care

Some patients expect “Internet + Chinese medicine” home care in the implementation of the process of nursing staff can be based on the actual situation of the patient’s condition dialectical care, and enjoy quality care services. N14: “If traditional Chinese medicine home care can really care for the actual situation of the patient’s condition, the effect must be obvious.” N22: “Home care needs a good attitude and service, and the technology is slightly excellent. Because Chinese medicine itself is the treatment of chronic diseases and health care, the effect is slightly slower, if the technology is not good, you cannot see the effect.”

##### 3.2.3.3. The need to improve the health insurance and service system

The health insurance reimbursement system in the “Internet + nursing” field is still in the exploration stage, only some developed regions to carry out pilot projects, has not yet been fully promoted and implemented, the patient hopes that the health insurance will included the “Internet + Chinese medicine” home care field while constructing the formation of the “Internet + Chinese medicine” home care management network demand. N8: “I think it’s still medical insurance, and it would be nice if it could participate, but I think it would be a bigger percentage.” N17: “Because most of the older adult now have health insurance, home care and health insurance are linked, and home care can be reimbursed as part of the reimbursement.” N15: “I suggest that this must not be copied, but must be based on the needs, according to the characteristics of the local community, that is, it is necessary to establish a targeted, distinctive model in line with the national conditions, local characteristics of the model.” N16: “To form a management network, a service network, for ex ample, in this region you include all the outlets that can be involved, then the service is a little bit easier.”

## 4. Discussion

### 4.1. Strengthening the demand for care and enhancing facilitating factors

The results of the quantitative study showed that 72.7% of the respondents were willing to receive home care services from nurses, and it was also understood through interviews that patients had a positive attitude towards it, similar to the results of previous studies (27, 28). This indicates that patients have a high demand for “Internet + TCM” home care. The reason for this may be related to the fact that “Internet + TCM” nursing can enable patients to enjoy high-quality medical services conveniently and optimize the allocation of medical resources.

Perceived usefulness refers to the extent to which users believe that the application system can improve their work efficiency, and the perceived usefulness of the application system (29), which can motivate patients to be more willing to participate in the home-based model. Some studies show that perceived usefulness has a positive impact on patients’ attitudes toward participating in “Internet + Nursing,” and the more patients pay attention to the actual effects of “Internet + Nursing,” the stronger their willingness to use it (30, 31). In addition, due to the diseases of older adult patients with chronic diseases, the use of Internet + Nursing has a positive impact on their attitude toward the use of Internet + Nursing. In addition, due to the characteristics of the diseases of older adult chronic disease patients, if they do not have effective management, the disease will worsen and deteriorate (32). Therefore, the penetration and integration of Chinese medicine technology and home care ensures the continuity and timeliness of services, and the home can enjoy professional nursing services, which significantly improves the quality of life of such patients. Along with the progress of Internet technology, home care will gradually become the development trend of medical service industry (33, 34). The home care service model of TCM can effectively meet the nursing needs of older adult patients with chronic diseases, and further research should focus on the home care model to solve the needs of older adult patients (35).

Therefore, it is suggested that the medical staff should continuously improve the knowledge quality and cognitive level of patients. From the perspective of cultural literacy of Chinese medicine, help the patients to form a correct understanding of the concepts and ideas of Chinese medicine, which in turn promotes the promotion and application of traditional Chinese medicine technology and the development of the Chinese medicine healthcare industry.

### 4.2. Improve the service system and overcome obstacles factors

The results of the quantitative multifactorial analysis study indicate that the cognitive level of TCM knowledge of the older adult chronic disease population will affect the nursing needs of traditional Chinese medicine. From the scores of the knowing items, it can be understood that the older adult chronic disease has a low knowledge of TCM and stays at the level of the therapeutic tools (36). In the qualitative study, it was also shown that the interviewed patients had only a superficial understanding of the “Internet + Chinese medicine” home care service model, but did not have an in-depth understanding of it, and some of the patients said that they had not understood this model. The reason for this may be related to the short period of time since the introduction of the “Internet + Nursing” policy, and the fact that medical institutions in the region have not yet launched the corresponding service system, which is similar to the results of study of Thomas (37). The results of the quantitative multifactorial analysis of this study showed that cell phones and television in the “Internet + Nursing” service pathway affect the demand for Chinese medicine care for older adult patients with chronic diseases, which is consistent with the qualitative results.

Digital literacy refers to patients’ access to and ability to use computers and the Internet (38). This indicates that patients have little access to the Internet and have limited ability to use mobile phones, computers, and other electronic devices, resulting in a low level of digital literacy of patients, which hinders the willingness of some patients to participate in “Internet + traditional Chinese medicine” home care services. It may also be related to physiological decline, cognitive and learning decline and economic factors in older adult patients with chronic diseases, which is consistent with the findings of the study of Ha (39). In addition, the quantitative study showed that the highest concern of older adult patients with chronic diseases about “Internet + Nursing” home care service was the high cost of the service (46.8%), which was consistent with the results of the qualitative study. The interviewed patients indicated that they needed to consider the cost of the service, and that there was a demand for the inclusion of health insurance in this model. The reason may be that the willingness to use is related to the value and price of this model, and patients’ willingness to use is affected by online diagnosis and treatment costs, medical insurance reimbursement, etc. Patients will consider the comparison of online and offline medical treatment costs, and the category and proportion of medical insurance reimbursement (40). The United States, Japan, and European countries and other countries have formed a more mature and distinctive home care service model (41, 42). While China’s Internet + home care service is still in its infancy, there are limitations in the content and scope of services.

It is necessary to strengthen the publicity of “Internet + Chinese medicine” home care services, carry out diversified, multi-channel, online and offline publicity methods, improve patients’ awareness of “Internet + Chinese medicine” home care, enhance social influence, and change patients’ inherent medical habits. And the creation and improvement of the “Internet + traditional Chinese medicine” home care service model has a practical role. It is suggested that medical institutions should, according to the national situation, start from the policy of home service, service cost, reimbursement system, and other aspects, in order to provide guarantee for the development of “Internet + Chinese medicine” home care service model suitable for older adult patients with chronic diseases.

### 4.3. Improve service quality and provide precise TCM care

The results of this study show that older adult patients with chronic diseases hope that “Internet + Chinese medicine” home care can enjoy targeted, high-quality, integrated Chinese and Western medicine nursing services. Nurses in the home care service need to have the ability to provide high-quality “Internet + Chinese medicine nursing service” (43). Research has confirmed that the dialectical and holistic concepts of TCM nursing techniques have the advantage of improving the health status of older adult patients with chronic diseases from a holistic perspective, promoting recovery and alleviating pain, and facilitating dialectical nursing care from a wide range of diseases (44). At the same time, quality care emphasizes people-oriented, providing patients with a full range of nursing services, in line with the holistic concept of Chinese medicine nursing and people-oriented ideology, the integration of appropriate technology of Chinese medicine nursing into quality nursing services can not only improve the quality of nursing services, but also accelerate the promotion of the culture of traditional Chinese medicine (45). In addition, studies have shown that Chinese and Western medicine culture can be promoted faster. Studies have shown that integrated Chinese and Western medicine care can provide patients with multifaceted care, regulate their physical and psychological conditions, improve their health awareness and clinical symptoms (46, 47).

It can be seen that we should strengthen the training of nursing staff, invite Chinese medicine nursing experts to organize overall training, teachers and students, carry out special lectures, Chinese medicine nursing knowledge and technology competitions. Encourage nurses to invest in research of TCM nursing and actively learn the characteristic nursing techniques of TCM. Through training, the ability to communicate and the ability to deal with emergencies will be enhanced, the dialectical thinking of nursing staff is cultivated, and the knowledge and skills of TCM nursing staff are improved, so as to provide patients with accurate, high-quality and diversified TCM nursing services.

### 4.4. Potential shortcomings and limitations

#### 4.4.1. Potential shortcomings

Both the medical association service model and home care services are in their infancy, and there are service team members with low professional quality, low motivation, and unclear division of labor. The service content is relatively single, not strong in specialty, unable to meet the needs of patients. Relevant supporting policies are not perfect, and there is a lack of unified and standardized service processes and standards.

#### 4.4.2. Limitations

First of all, this study only selected patients from one hospital in Xi’an due to manpower and time constraints, and the results of the study may have some regional limitations. In future studies, researchers can select several different regions to increase the sample size of the study and further explore the current situation and influencing factors of the demand for TCM home care for older adult patients with chronic diseases.

Second, this study used purposive sampling method for qualitative research, and its findings can only represent the experience of some patients and cannot be inferred to the whole. Random stratified sampling in different regions makes the study more rigorous.

Finally, this study used a combination of quantitative and qualitative research methods, and the two research methods complemented each other, while the qualitative research obtained new relevant factors not reflected in the quantitative part of this study through the deeply excavated information. Further relevant factors need to be added in future investigations.

## 5. Conclusion

In summary, although “Internet + Chinese medicine” home care service is in the exploratory stage, older adult patients with chronic diseases have higher demand for it and stronger willingness to participate in it, and it has a broad development prospect, but there are still many concerns. Medical institutions should strengthen publicity and promotion efforts to increase the social influence and participation of “Internet + Chinese medicine” home care services. Also, strengthen the training of nursing staff, comprehensively improve their comprehensive quality, provide patients with high-quality and diversified care programs. The government should strengthen support, improve the medical insurance system, increase capital investment, and build a more scientific, feasible and reasonable “Internet + traditional Chinese medicine” home care service model for older adult patients with chronic diseases. The survey respondents of this study are mainly gathered in a tertiary hospital, which may have a certain bias. The next step is to examine patients’ needs for home care services from all angles and in all aspects in different regions, so that the results of the study can be more representative.

## Data availability statement

The original contributions presented in the study are included in the article/**Supplementary material**, further inquiries can be directed to the corresponding author.

## Ethics statement

Written informed consent was obtained from the individual(s) for the publication of any potentially identifiable images or data included in this article.

## Author contributions

XW: Data curation, Formal analysis, Investigation, Validation, Writing – original draft, Writing – review & editing. JC: Data curation, Formal analysis, Writing – review & editing. MF: Writing – original draft. MZ: Software, Supervision, Writing – review & editing. JW: Data curation, Investigation, Writing – original draft. LZ: Formal analysis, Methodology, Writing – original draft. YL: Data curation, Investigation, Writing – original draft. HC: Methodology, Project administration, Resources, Supervision, Visualization, Writing – review & editing.
