# Supplementary material for: Demand and influencing factors of “Internet + Traditional Chinese Medicine” home nursing service for older adult patients with chronic diseases: a mixed research perspective
Source: Front Public Health. 2023 Oct 17;11:1271082. doi: 10.3389/fpubh.2023.1271082 (PMC10622788; doi:10.3389/fpubh.2023.1271082)
Supplement: Supplementary file 1 [file Table_1.DOCX]

Table 1: General information of respondents and one-way analysis of factors

| Item | Categories | Frequency | Percentage | Chinese Medicine Needs Score (score,x±s) | *T/F*-values | *P*-values |
| --- | --- | --- | --- | --- | --- | --- |
| Age (years) | 60-64 | 70 | 22.70% | 56.81±16.40 | 1.256^2）^ | 0.288 |
|  | 65-69 | 67 | 21.80% | 58.45±18.41 |  |  |
|  | 70-74 | 67 | 21.80% | 62.30±17.55 |  |  |
|  | 75-79 | 68 | 22.10% | 56.38±17.53 |  |  |
|  | ≥80 | 36 | 11.70% | 58.14±14.19 |  |  |
| Gender | male | 170 | 55.2% | 59.88±17.54 | 1.656^1）^ | 0.099 |
|  | female | 138 | 44.8% | 56.63±16.56 |  |  |
| Living condition | Living alone | 28 | 9.1% | 57.21±19.87 | 0.284^2）^ | 0.837 |
|  | Living with spouse | 193 | 62.7% | 59.07±17.74 |  |  |
|  | Living with children | 59 | 19.2% | 56.93±14.36 |  |  |
|  | Living with spouse and children | 28 | 9.1% | 58.32±16.17 |  |  |
| Marital status | Unmarried | 1 | 0.3% | 47.00±0.00 | 0.344^2）^ | 0.848 |
|  | Married | 274 | 89.0% | 58.44±17.47 |  |  |
|  | Remarried | 2 | 0.6% | 70.00±11.31 |  |  |
|  | Divorced | 1 | 0.3% | 57.00±0.00 |  |  |
|  | Widowed | 30 | 9.7% | 57.90±14.98 |  |  |
| Educational level | Illiterate | 9 | 2.9% | 60.56±14.82 | 0.833^2）^ | 0.602 |
|  | Elementary School | 41 | 13.3% | 59.88±12.28 |  |  |
|  | Junior high school | 79 | 25.6% | 55.47±20.35 |  |  |
|  | High School or Junior College | 109 | 35.4% | 59.62±15.31 |  |  |
|  | College and above | 70 | 22.7% | 58.76±18.65 |  |  |
| occupation | Farmer | 20 | 6.5% | 58.85±14.61 | 0.748^2）^ | 0.588 |
|  | Laborer | 10 | 3.2% | 62.80±13.94 |  |  |
|  | Institutions and enterprises | 6 | 1.9% | 57.50±12.13 |  |  |
|  | Unemployed | 8 | 2.6% | 51.25±5.68 |  |  |
|  | Retirees | 258 | 83.8% | 58.67±17.83 |  |  |
|  | Others | 6 | 1.9% | 49.50±12.44 |  |  |
| Percapita household income | ＜1500 | 22 | 7.10% | 61.73±14.99 | 1.172^2）^ | 0.346 |
|  | 1500-3500 | 135 | 43.80% | 56.52±19.75 |  |  |
|  | 3500-5000 | 103 | 33.40% | 60.14±15.54 |  |  |
|  | ≥5000元 | 48 | 15.60% | 58.58±12.80 |  |  |
| Medical insurance type | Basic medical insurance for urban workers | 259 | 84.1% | 58.09±17.30 | 0.596^2）^ | 0.666 |
|  | Basic Medical  Insurance for Urban  Residents | 22 | 7.1% | 61.27±19.09 |  |  |
|  | New Rural Cooperative Medical Care | 25 | 8.1% | 60.20±14.12 |  |  |
|  | Other Social Medical Insurance | 1 | 0.3% | 57.00±0.00 |  |  |
|  | Self-financed | 1 | 0.3% | 38.00±0.00 |  |  |
| Number of chronic diseases | 1 type | 82 | 26.6% | 67.30±15.58 | 12.313^2）^ | ＜0.001 |
|  | 2 types | 158 | 51.3% | 55.82±16.27 |  |  |
|  | 3 types | 64 | 20.8% | 52.92±16.90 |  |  |
|  | More than 3 types | 4 | 1.3% | 67.25±21.59 |  |  |
| Primary caregiver | Spouse | 202 | 65.6% | 59.49±16.83 | 1.227^2）^ | 0.300 |
|  | Child | 90 | 29.2% | 56.98±17.56 |  |  |
|  | Caregiver | 2 | 0.6% | 62.50±2.12 |  |  |
|  | Others | 14 | 4.5% | 51.71±19.47 |  |  |
| Whether the caregiver can meet the care needs | Fully satisfy  Can basically satisfy  Cannot satisfy  Can't satisfy at all | 40 | 13.0% | 47.75±16.08 | 6.441^2）^ | ＜0.001 |
|  | Can basically satisfy | 229 | 74.4% | 59.73±17.24 |  |  |
|  | Cannot satisfy | 36 | 11.7% | 61.97±13.88 |  |  |
|  | Can't satisfy at all | 3 | 1.0% | 58.33±14.22 |  |  |
| Educational activities in the community | Often | 20 | 6.5% | 60.50±10.17 | 0.555^2）^ | 0.449 |
|  | Occasionally | 141 | 45.8% | 57.35±16.45 |  |  |
|  | never | 147 | 47.7% | 59.16±18.54 |  |  |
| Knowledge of home care level | Don't understand | 201 | 65.3% | 53.38±15.76 | -7.716^1）^ | ＜0.001 |
|  | Have a certain understanding | 107 | 34.7% | 67.90±15.65 |  |  |
| How do you know about Internet + Nursing Home Care (multiple choice) | Newspapers, television | 57 | 18.5% | 62.65±13.47 | -2.461^1）^ | 0.015 |
|  | Cell phone or computer | 65 | 21.1% | 67.74±14.21 | -5.128^1）^ | ＜0.001 |
|  | Hospital publicity | 24 | 7.8% | 57.50±17.35 | 0.274^1）^ | 0.748 |
|  | Community publicity | 39 | 12.7% | 59.82±12.31 | -0.712^1）^ | 0.479 |
|  | Recommended by family or friends | 26 | 8.4% | 58.85±13.49 | -0.163^1）^ | 0.872 |
|  | Others (please specify) | 159 | 51.6% | 52.87±17.11 | 6.219^1）^ | ＜0.001 |
| Concerns about nursing home service (multiple options) | Personal safety is not high | 89 | 28.9% | 55.90±10.53 | 2.078^1）^ | 0.039 |
|  | Higher service cost | 144 | 46.8% | 55.18±15.70 | 3.152^1）^ | 0.002 |
|  | Nursing operation is risky | 109 | 35.4% | 53.50±11.79 | 4.333^1）^ | ＜0.001 |
|  | Personal privacy disclosure | 52 | 16.9% | 55.96±11.02 | 1.556^1）^ | 0.122 |
|  | Nurses have a heavy workload | 38 | 12.3% | 54.74±10.74 | 2.048^1）^ | 0.044 |
|  | Others (please specify) | 89 | 28.9% | 64.99±22.78 | -3.580^1）^ | 0.001 |
| Whether you would like home care services | Willing | 224 | 72.7% | 61.01±17.78 | 12.544^2）^ | ＜0.001 |
|  | Uncertain | 63 | 20.5% | 53.86±9.01 |  |  |
|  | Unwilling | 21 | 6.8% | 44.52±19.98 |  |  |
| 1)*T*-values；2)*F*-values。 | | | | | | |
